# Supplementary material for: Chromothripsis during telomere crisis is independent of NHEJ, and consistent with a replicative origin
Source: Genome Res. 2019 May;29(5):737–49. doi: 10.1101/gr.240705.118 (PMC6499312; doi:10.1101/gr.240705.118)
Supplement: Supplemental Material [file supp_gr.240705.118_Supplemental_file_1.zip › contigs/annotated_contigs/DB46/contig.2.DB46_length_538_mean_cov_4.16728624535.docx]

**DB46_length_538_mean_cov_4.16728624535**

```
AGAGTGACCATGGGTTCCAAAGGAGGGTTAGACAGAATGCCAAACAATGGGACAAACAAGAGGAGTCTGAAGAGATTTCCTGGGAATCT
 >chr1:52434433-52434651 - E=2e-113 p=3e-02
GAATTCCACAGTGATGTGAGTAACTCCTCATATAAACAGGAACTGAGAACAAGGTCAAGGTGAGATGGGGTGCTGGGCAGTCAGCAAGG

TTCGCTTATCAGTCATCTGCCTTATGTCCACACAGCAG|CC|CAGTTACTTGAGAGGCTGAGGTGGGAGGATCACTTGAGCCTGGGAAT
                                       >chr1:52405060-52405382 - E=4e-176
TTGAGGCTGCAGTGAGCTGTGATCATGCACTCAGGACTGGATGACAGCGTGACATCCTGTCTCAAAACAAAAAAAGCTGGGTACACTGG

CGAGCGCCTGTAGTCCTAGCTATTCTGCAGGCTGAGGTGGGAGGATCACTTGAGCCTAGGAGTTCAAGGCTGCAGTGAGCTATGATGAT

TGTGCCACTGCTCTCTAGCTTGAGTGACAGAGTGAGACCCTGTCTAATAAAAAAAAAAAGTCCCAGATTTAAGGCCTACCTTTTTGTCA

CCAGCT
```
